# Predictive models of adverse outcomes of overweight or obesity: a nationwide cohort validation

**DOI:** 10.1186/s12902-026-02399-4

**Published:** 2026-07-10

**Authors:** Alexander Turchin, Maria Shubina, Fritha J. Morrison, Nadia N. Ahmad, Lisa M. Neff, Hong Kan

**Affiliations:** 1 Brigham and Women’s Hospital, Boston, MA USA; 2 Harvard Medical School, Boston, MA USA; 3 Eli Lilly and Company, Indianapolis, IN USA

**Keywords:** Obesity, Overweight, Predictive modeling, Long-term outcomes, Validation

## Abstract

**Background:**

Obesity and overweight have a wide range of adverse clinical outcomes. Predictive models could help clinicians identify individuals with obesity and overweight at greater risk for obesity-related complications, enabling personalized shared decision-making about obesity treatment. We previously developed a set of predictive models of adverse clinical outcomes of obesity and overweight using electronic health record (EHR) data of a large health system in Massachusetts. However, these models have not been validated on data from other sources.

**Methods:**

We validated 12 predictive models for cardiometabolic, oncologic and musculoskeletal complications of excess weight using a linked EHR and claims dataset of a national patient cohort (Optum^®^ Market Clarity) that included adults 18–80 years of age with body mass index (BMI) ≥ 25 kg/m^2^ between 2007 and 2023.

**Results:**

A 5% randomly selected validation dataset included 437,223 patients (51.9% women) with a mean age of 51.4 years and a mean BMI of 31.9 kg/m^2^ who were followed for a mean of 5.7 years. Most (11/12) models achieved Harrell C-index ≥ 0.70. Most (11/12) models had either 5-year or 10-year calibration slope within 0.3 of the ideal value of 1.0; 7 out of 12 had either 5-year or 10-year calibration slope that was not significantly different from 1.0; all had intercepts very close to 0.

**Conclusions:**

A set of EHR-based predictive models of adverse outcomes of obesity and overweight was generalizable to a large national cohort. These findings suggest potential clinical and population health applications for predictive models in treatment of individual patients with obesity / overweight, as well as in population management and quality improvement.

**Clinical trial number:**

Not applicable.

**Supplementary Information:**

The online version contains supplementary material available at https://doi.org/10.1186/s12902-026-02399-4.

## Background

Obesity is one of the most common chronic diseases in the U.S [1]. Individuals with overweight or obesity are at an increased risk for multiple adverse outcomes, ranging from coronary artery disease and stroke to diabetes mellitus, multiple types of cancer, liver disease and death [2]. Lifestyle interventions, pharmacotherapy and metabolic / bariatric surgery can (to a variable degree) reduce excess adiposity and decrease the risk of future complications [3–6]. On the other hand, patients and clinicians face a number of barriers to implementation of these interventions, including limited time and resource in clinic [7, 8]; challenges with insurance coverage and cost [4, 9–11], and possible concerns about adverse events [12–15]. Consequently it can be helpful to be able to identify patients who are most likely to benefit from the intervention in order to inform shared decision-making and direct limited healthcare resources. This could be accomplished using predictive models that can evaluate the risk of obesity-related complications. While several predictive models for individual obesity-related complications exist [16, 17], for many of these complications there are no predictive models that incorporate measures of the patient’s weight; and few of the published risk prediction models for obesity-related complications were specifically developed in people with overweight or obesity. It is also important to ensure that predictive models being considered for clinical use are generalizable by evaluating them on a broad population sample not used to derive the original model [18, 19].

Growing availability of electronic health records (EHR) data provides an opportunity to assess adverse clinical outcomes and their risk factors across large populations over extended periods of time [20, 21]. We previously leveraged a large EHR dataset to develop a series of high-performance risk prediction models for long-term obesity-related complications [22]. We have now conducted a study to externally validate these predictive models on a national dataset of EHR and health insurance claims data.

## Methods

### Study design

A series of 12 predictive models of adverse clinical outcomes of overweight or obesity that were originally developed using EHR data of a single large healthcare delivery system were externally validated using retrospective EHR and health insurance claims data for a nationwide patient cohort.

### Study cohort

Study participants included adults (age ≥ 18 years) with body mass index (BMI) between 25 and 80 kg/m^2^ who had both EHR and claims data in the Optum^®^ Market Clarity database between 2007 and 2023. Patients were excluded from the study if they: (a) were older than 80 years old; (b) had missing information on biological sex; (c) did not have estimated glomerular filtration rate (eGFR) measurements available or (d) were diagnosed with a disease outcome of interest at baseline. The baseline disease outcome diagnosis exclusion criterion was applied on a per-analysis basis (e.g. patients with history of cancer were only excluded from the analysis where incidence of cancer served as the outcome). A randomly selected 5% subset of eligible patients was used for analysis to optimize time and hardware requirements of statistical calculations.

### Study measurements

A patient was entered into the study (*Index Date*) on the first date on or after *Study Start* date (01/01/2007) when they met all of the following criteria: (1) had data available for ≥ 12 months prior to the Index Date (to ascertain baseline patient characteristics); (2) were ≥ 18 years old; and (3) had first of two consecutive BMI measurements ≥ 25 kg/m^2^. Patients exited the study (*Study Exit Date*) on the first of the following dates: (a) reaching an outcome endpoint (this criterion was specific to the outcome being analyzed); (b) death (even when it is not a component of the outcome being analyzed); (c) when data was no longer available 24 months after the last primary care note (i.e. lost to follow-up) or (d) *Study End* (12/31/2023). Patients were enrolled into the study until 12/31/2022 (to allow at least 12 months of follow-up to assess study outcomes).

Patients’ baseline characteristics and outcomes were obtained from Optum^®^ Market Clarity database. Optum^®^ Market Clarity links EHR data from multiple healthcare delivery organizations across the U.S. with health insurance claims information, including pharmacy claims, physician claims and facility claims. It includes patients in all 50 U.S. states.

The following endpoints were assessed: (a) atherosclerotic cardiovascular disease (ASCVD); (b) heart failure (HF); (c) diabetes mellitus type 2 (T2D); (d) chronic kidney disease (CKD); (e) metabolic dysfunction-associated steatohepatitis (MASH) / metabolic dysfunction-associated steatotic liver disease (MASLD); (f) obstructive sleep apnea; (g) all cancers (excluding non-melanoma skin cancer); (h) obesity-associated cancers; (i) degenerative joint disease (DJD); (j) knee replacement and k) all-cause mortality. The following malignancies were included in the obesity-associated cancers outcome: biliary, breast, colorectal, endometrial, esophageal, gastric, kidney, liver, ovarian, pancreatic, multiple myeloma and meningioma. Breast cancer and cancers of female reproductive organs were only included in the obesity-associated cancer outcome for women and the obesity-associated cancer outcome was calculated separately for women and men. Length of time (days) from the Index Date to each of the clinical events listed above served as study outcomes.

All variables were assessed at baseline, defined as the most recent record / measurement prior to study entry. For quantitative variables, if no measurements prior to study entry were available, the earliest measurement within one month after study entry was used. eGFR was calculated using equations that did not include race [23]. Missing HbA1c measurements were imputed using random draws from normal distributions based on published data for similar populations, separately for patients with (mean 8.3% and SD 1.7%) versus without (mean 5.6% and SD 0.7%) diabetes, as previously described [22]. Medical terminology codes used to identify candidate predictor variables and study outcomes are provided in Supplemental Table 1 Sources of information for study outcomes are provided in Supplemental Table 2 Definitions of all outcome endpoints and baseline predictor variables were identical between the derivation (described in detail in the original publication [22]) and the external validation (the present analysis) cohorts.

### Statistical analysis

The original predictive models were developed using EHR data from Mass General Brigham (MGB) integrated healthcare delivery system as previously described [22]. Variables included in each model are provided in Table 1. These predictive models were constructed using Fine-Gray subdistribution proportional hazards models [24] with all-cause death as competing risk event. Cox proportional hazards models were used for the all-cause mortality outcome.

**Table 1:** Variables included in predictive models of adverse clinical outcomes of overweight or obesity

Variable	ASCVD	HF	T2D	CKD	OSA	MASH / MASLD	CA: Any	CA: Obesity-associated		DJD	Knee replacement	Death
								Women	Men			
BMI	X	X	X	X	X	X	X	X	X	X	X	X
Age	X	X	X		X		X	X	X	X	X	X
Sex	X				X					X		
Smoking status	X					X	X			X		
Alcohol abuse									X			
Family history of cancer					X	X	X	X				
Family history of diabetes			X		X	X						
Family history of ASCVD					X							
ASCVD		X										X
Heart failure				X								X
T2D	X	X		X		X			X			
Cancer												X
DJD											X	
Hypertension	X	X	X	X		X			X	X		
Valvular heart disease		X										
Knee injury										X	X	
Hepatitis B												X
Hepatitis C									X			
Chronic inflammation										X		
Proteinuria				X								
eGFR	X	X		X								X
HbA1c			X	X		X						

ASCVD: atherosclerotic cardiovascular disease; BMI: body mass index; CA: cancer; CKD: chronic kidney disease; DJD: degenerative joint disease; eGFR: estimated glomerular filtration rate; HF: heart failure; MASLD: metabolic dysfunction-associated steatotic liver disease; MASH: metabolic dysfunction-associated steatohepatitis; OSA: obstructive sleep apnea; T2D: type 2 diabetes mellitus

For each study outcome we evaluated both discrimination and calibration performance. Discrimination was evaluated by calculating Harrell concordance index [25] (C-index) for each predictive model (i.e. each of the 12 outcomes) on the Optum^®^ Market Clarity dataset and comparing it to the Harrell C-index calculated for the same model on the original (20%) held-out validation dataset derived from MGB EHR data. Calibration was evaluated at 5- and 10-year time points, averaging predicted and observed cumulative incidence function / risk values by these groups by deciles of predicted values, and estimating regression intercepts and slopes of the averaged observed values on the averaged predicted values. The null hypothesis of the calibration slope being equal to 1.0 was tested using the standard error estimate for the slope from the regression model. Similarly, the null hypothesis of the calibration intercept being equal to 0 was tested using the standard error estimate for the intercept from the regression model. All statistical analyses were performed using R version 4.3.3 (R Foundation for Statistical Computing, Vienna, Austria).

## Results

### Study cohort

**Fig. 1:**
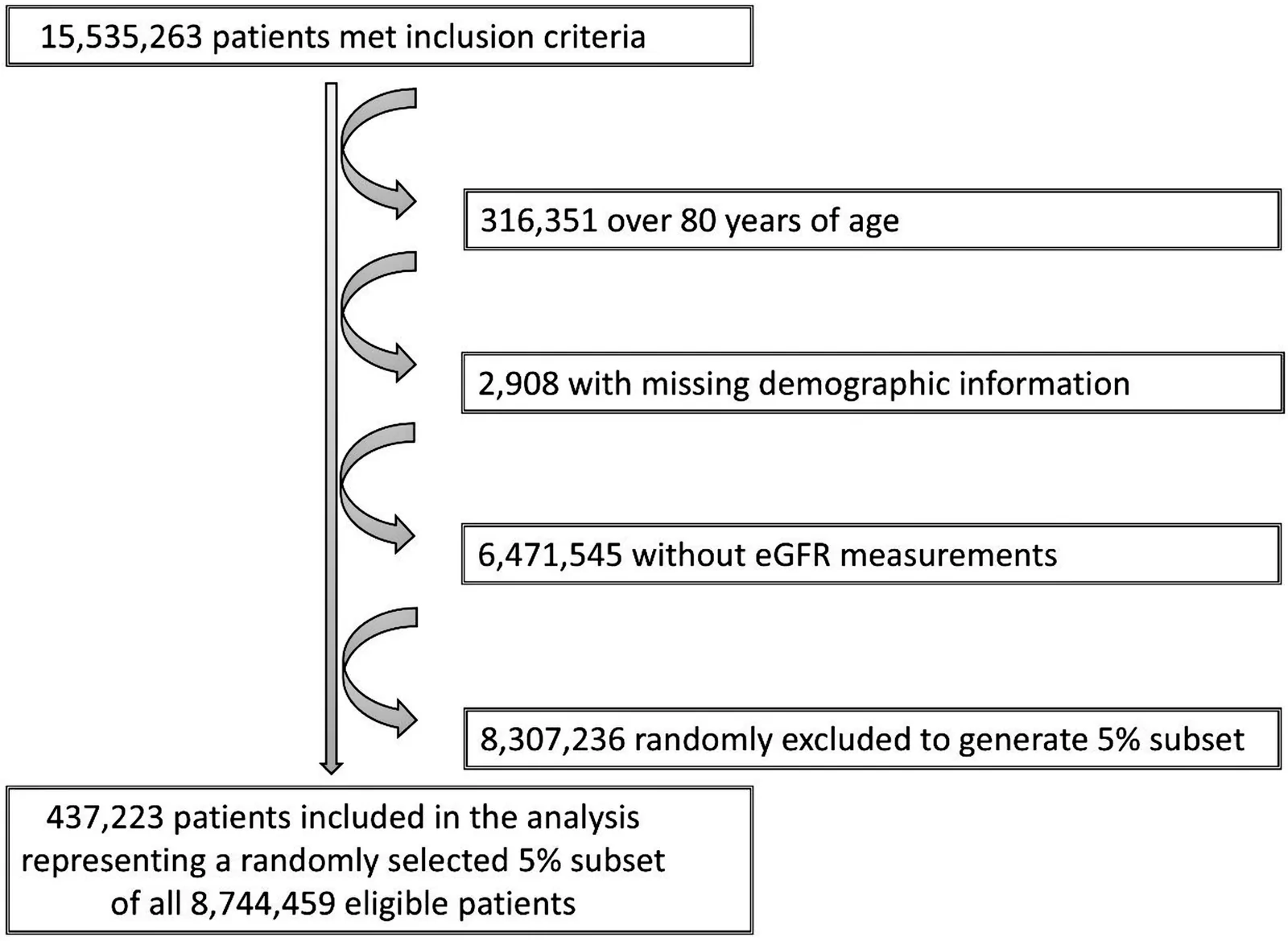
Patient flow chart. eGFR: estimated glomerular filtration rate

A total of 15,535,263 adults with BMI between 25 and 80 kg/m^2^ who had baseline data in Optum^®^ Market Clarity were identified. After excluding patients who (a) were older than 80, (b) did not have biological sex information available or (c) did not have eGFR measurements available, 8,744,459 patients fulfilling study eligibility criteria were identified. Out of these, a random 5% subset of 437,223 patients was included in the analysis (Fig. 1). Compared to the patients in the dataset used to derive the original predictive models, patients in the external validation dataset (Table 2) were older (mean age 51.4 vs. 47.9 years in the external validation vs. derivation datasets) and had higher weight (mean BMI 31.9 vs. 30.5 kg/m^2^); a similar proportion were female (51.9% vs. 52.2%) and white (78.8% vs. 75.0%). Over the mean follow up time of 2,096 days (5.7 years), incidence rate of study outcomes ranged from 0.40 per 100 patient-years for obesity-associated cancers among men to 3.1 per 100 patient-years for ASCVD (Table 3). Incidence of most study outcomes was slightly higher in the external validation compared to the derivation dataset (Table 3), as expected in an older population with a higher BMI.

**Table 2:** Baseline characteristics of study patients

Variable	Mean (SD) or *N* (%)	Missing, *N* (%)
Age, years	51.4 (15.9)	0
Female	226,814 (51.9)	0
Race		
Asian	8,303 (1.9)	
Black	51,374 (11.8)	
White	344,395 (78.8)	
Other (includes unknown)	33,151 (7.6)	
Hispanic ethnicity	24,407 (5.6)	
Smoking history	265,464 (60.7)	0
Alcohol abuse	10,342 (2.4)	0
ASCVD	51,597 (11.8)	0
Heart failure	17,399 (4.0)	0
T2D	65,440 (15.0)	0
MASH / MASLD	7,233 (1.7)	0
Obstructive sleep apnea	26,713 (6.1)	0
Cancer	27,976 (6.4)	0
Obesity-associated cancer	12,595 (3.0)	0
DJD	81,609 (18.7)	0
Knee replacement	5,747 (1.3)	0
Proteinuria	10,541 (2.4)	0
Hepatitis B	871 (0.2)	0
Chronic inflammation^1^	10,704 (2.4)	0
Knee injury	15,750 (3.6)	0
Valvular heart disease	11,201 (2.6)	0
Hypertension	210,213 (48.1)	0
Family history of ASCVD	44,154 (10.1)	0
Family history of diabetes	59,238 (13.5)	0
Family history of cancer	58,808 (13.5)	0
BMI, kg/m^2^	31.9 (6.5)	0
HbA1c, %	6.5 (1.7)	300,282 (68.7)
LDL-C, mg/dL	110 (36)	162,715 (37.2)
eGFR, mL/min/1.73 m^2^	88 (23)	0

^1^Chronic inflammation incudes: (a) inflammatory bowel disease; (b) rheumatoid arthritis; (c) systemic lupus erythematosus; and (d) multiple sclerosis

The table represents baseline characteristics of the study population before any outcome-specific exclusion criteria were applied (i.e. the study population used for external validation of the all-cause mortality predictive model)

ASCVD: atherosclerotic cardiovascular disease; BMI: body mass index; DJD: degenerative joint disease; eGFR: estimated glomerular filtration rate; LDL-C: low density lipoprotein cholesterol; MASLD: metabolic dysfunction-associated steatotic liver disease; MASH: metabolic dysfunction-associated steatohepatitis; T2D type 2 diabetes mellitus

**Table 3:** Incidence of study outcomes

Outcome	Study Population^1^	Incidence Rate^2^: MC	Incidence Rate^2^: MGB
ASCVD	385,626	3.1	1.7
Heart failure	419,824	1.4	0.70
T2D	371,783	2.6	1.5
CKD	434,059	0.36	0.31
MASH / MASLD	416,433	0.85	0.65
OSA	410,510	2.2	1.3
CA: all	409,247	1.5	1.3
CA: obesity-associated			
Women	210,375	0.73	0.66
Men	197,705	0.40	0.40
DJD	355,614	2.2	2.0
Knee replacement	431,476	0.43	0.25
All-cause mortality	437,223	1.4	0.66

^1^In the present study (Optum^®^ Market Clarity dataset)

^2^Per 100 person-years

ASCVD: atherosclerotic cardiovascular disease; CA: cancer; CKD: chronic kidney disease; DJD: degenerative joint disease; MASLD: metabolic dysfunction-associated steatotic liver disease; MASH: metabolic dysfunction-associated steatohepatitis; MC: Market Clarity; MGB: Mass General Brigham; OSA: obstructive sleep apnea; T2D: type 2 diabetes mellitus

### Predictive model validation

Out of 12 predictive models evaluated in this study, 11 achieved Harrell C-index of 0.70 and above on the external validation dataset (Table 4). The only exception was the predictive model for MASLD / MASH that remained only modestly discriminative and achieved a C-index of 0.65 – similar to its original evaluation on the derivation dataset [22].

**Table 4:** Predictive model validation

Outcome	Harrell C-Index	Calibration: 5 years		Calibration: 10 years	
		Slope (*P*-value^1^)	Intercept (*P*-value^2^)	Slope (*P*-value^1^)	Intercept (*P*-value^2^)
ASCVD	0.78	1.29 (< 0.001)	-0.003 (0.71)	1.08 (0.23)	0.011 (0.52)
Heart failure	0.82	1.36 (< 0.001)	0.003 (0.63)	1.12 (0.19)	0.014 (0.28)
T2D	0.77	1.50 (< 0.001)	-0.003 (0.73)	1.18 (0.036)	0.013 (0.35)
CKD	0.83	1.05 (< 0.001)	-0.0012 (< 0.001)	0.80 (< 0.001)	0.0014 (0.39)
MASH / MASLD	0.65	0.97 (0.81)	0.010 (0.11)	0.79 (0.09)	0.022 (0.026)
OSA	0.70	1.54 (< 0.001)	0.006 (0.34)	1.24 (0.002)	0.027 (0.008)
CA: all	0.72	1.16 (< 0.001)	-0.006 (0.052)	0.92 (0.11)	0.004 (0.59)
CA: obesity-associated					
Women	0.75	1.01 (0.91)	-0.0006 (0.88)	0.79 (0.047)	0.006 (0.46)
Men	0.72	0.81 (0.003)	0.0002 (0.87)	0.66 (< 0.001)	0.004 (0.29)
DJD	0.71	0.93 (0.20)	-0.003 (0.70)	0.77 (0.003)	0.015 (0.32)
Knee replacement	0.83	1.12 (0.13)	-0.0018 (0.38)	1.03 (0.76)	0.0013 (0.78)
All-cause mortality	0.83	1.78 (< 0.001)	0.0007 (0.86)	1.79 (< 0.001)	0.011 (0.54)

^1^For difference from 1.0

^2^For difference from 0.0

ASCVD: atherosclerotic cardiovascular disease; CA: cancer; CKD: chronic kidney disease; DJD: degenerative joint disease; MASLD: metabolic dysfunction-associated steatotic liver disease; MASH: metabolic dysfunction-associated steatohepatitis; OSA: obstructive sleep apnea; T2D: type 2 diabetes mellitus

In the evaluation of 5-year calibration, 4 out of 12 predictive models (MASH / MASLD, female obesity-related cancers, DJD and knee replacement) had a calibration slope that was not significantly different from 1.0 (representing a perfectly accurate prediction of outcome probability at 5 years across all levels of predicted risk) and 8 out of 12 predictive models had calibration slope in the 0.70–1.30 range (Table 4), representing acceptable calibration. The lowest 5-year calibration slope was for the male obesity-associated cancers predictive model at 0.81 and the highest for the all-cause mortality predictive model at 1.78. Intercepts for all predictive models in 5-year calibration were between − 0.01 and 0.01 and only one was significantly different from 0 (representing no systematic under- or overestimation of risk). In the evaluation of 10-year calibration, 5 out of 12 predictive models (ASCVD, heart failure, MASH / MASLD, all cancers and knee replacement) had a calibration slope that was not significantly different from 1.0 to 10 out of 12 predictive models had calibration slope in the 0.70–1.30 range (Table 4). The lowest 10-year calibration slope was for the male obesity-associated cancers predictive model at 0.66 and the highest for the all-cause mortality predictive model at 1.79. Intercepts for all predictive models in 10-year calibration were between 0 and 0.03 and 10 out of 12 were not significantly different from 0.

## Discussion

In this study we present the results of a comprehensive external validation of a set of EHR data-derived predictive models for a broad range of adverse outcomes of overweight or obesity on a large nationwide patient cohort. Most predictive models performed well, demonstrating the potential of EHR data for assessment of patients’ risk of long-term clinical outcomes.

Other predictive models for some of the clinical outcomes examined in this study have previously been published [17, 26–30]. However, none of these models focused on the population of patients with overweight or obesity. Importantly, previously published studies have demonstrated that existing predictive models that were developed for the general population perform poorly in individuals with obesity [31]. Many of these models did not include BMI as a risk factor. The suite of predictive models validated in this study therefore represents an important advancement in our understanding and quantification of long-term risks of overweight or obesity.

An essential step in development of a predictive model is its external validation on a dataset that was drawn from a population different from the one used to derive the original model. This serves to ensure the model’s generalizability to other populations and therefore its usability for clinical decision making [32–34] because apparent model performance in the derivation cohort can reflect overfitting, local practice patterns, or database-specific data structure rather than true transportability. Failure of predictive models already in clinical practice that were ultimately found not to be applicable to the general population has been described [35]. At the same time, the majority of published predictive models are never externally validated [36]. When predictive models are externally validated, many exhibit a noticeably lower accuracy [36–38], possibly due to the high risk of bias inherent in their design procedures [39]. Importantly, most models evaluated in this study demonstrated a minimal decrease in performance metrics, such as C-index, when assessed on a nationwide cohort – a finding that indicates a high potential for clinical usability.

In addition to metrics evaluating model discrimination, such as C-index, another important aspect of model assessment is calibration, representing the accuracy of the model’s estimates of absolute risk. Despite its obvious role in evaluating clinical utility of the model, many external validations of predictive models do not include any measure of calibration [40]. In the present study, 11 out of 12 models had either 5-year or 10-year calibration slope within 0.3 of the ideal value of 1.0, indicating that the adverse outcome probabilities predicted by the model did not deviate by more than 30% (in either direction) from those observed in the external validation data. Additionally, 7 out of 12 models had either 5-year or 10-year calibration slope that was not significantly different from 1.0; and all had intercept very close to 0. One exception was the predictive model for all-cause mortality, which underestimated the risk in the validation Optum^®^ Market Clarity dataset. One possible explanation for this discrepancy is that incidence of all-cause mortality was higher in the Optum^®^ Market Clarity dataset than in the original study. Notably, the original model was developed in Massachusetts, which has one of the lowest mortality rates in the U.S [41]. The original predictive model for all-cause mortality may therefore need to be re-calibrated in a future study to better reflect mortality rates in the overall U.S. population.

A broad collection of validated high-performance predictive models described in this study could be used to improve patient care in a variety of ways. At the individual level it could be used to identify patients likely to reap the greatest benefit from treatment of their obesity or overweight. At the organizational level they could be employed to estimate expected changes in demand in specific clinical services and direct resource allocation accordingly. A health system could utilize these models to guide population management and value-based care programs. A large employer could take advantage of them to inform obesity care initiatives to reduce absenteeism and improve employee productivity.

In addition to serving as indicators of potential benefits of treatment of obesity and overweight, models assessed in this analysis could have clinical applications specific to the individual outcomes they focus on. For example, patients predicted to have a particularly high risk of ASCVD may be recommended more aggressive cholesterol-lowering therapy [42]; patients predicted to have a high risk of type 2 diabetes could be referred to a diabetes prevention program [43]; and patients predicted to have an elevated risk of cancer could increase frequency of established monitoring modalities (e.g. mammograms or colonoscopies) or perhaps even consider multi-cancer detection tests [44]. As with obesity therapy, clinical efficacy and cost-effectiveness of outcome-specific care changes based on model predictions would need to be evaluated before they can be broadly recommended.

It is now well established that obesity is a chronic disease with complex underlying pathophysiology [45]. At the same time many clinical and public health applications require an analytical model of obesity as a continuous and scalable risk factor. The present study therefore adopts a dual framework in which obesity is recognized as a chronic disease entity, consistent with prevailing clinical and public health perspectives, while also using BMI as its numerical representation. Limitations of BMI as a quantification of the pathophysiological mechanisms of excess adiposity are well known and it was chosen for our predictive models primarily because of its wide availability in patient care data. As the clinical paradigm continues to shift and more comprehensive assessments of obesity (e.g. including measurement of waist circumference, waist-to-height ratio, or body composition) are adopted for use in patient care, analytical approaches, including the models presented in this study, will need to be updated to incorporate them.

The present study has a number of strengths. It validated predictive models on a nationwide cohort of patients, increasing generalizability of its findings. A large dataset – over 400,000 patients – allowed for a statistically robust analysis. Finally, the models validated in this study focus on obesity, for which there is increasing recognition of its contribution to a wide range of adverse clinical outcomes and reduced quality of life.

Findings of this study should be interpreted in light of its limitations. The analysis utilized data that was not originally collected for research, and therefore it is possible that some patient data was missing and / or subject to bias. The study period spanned 17 years, during which substantial changes occurred in obesity management, diagnostic criteria, and coding practices. This temporal heterogeneity could be a potential source of bias affecting model performance. In particular, modern medications (e.g. glucagon-like peptide-1 receptor agonists and sodium-glucose cotransporter-2 inhibitors) that are commonly used by patients with obesity and may affect some of the outcomes studied were not included in the models. Some of the other patient characteristics that may affect model outcomes were not included to minimize computational load (e.g. temporal variables, such as weight trajectory) or due to their limited availability in EHR data (e.g. care access). Some of the models (e.g. 5-year prediction of T2D and OSA and both 5-year and 10-year prediction of all-cause mortality) had calibration slopes that indicated lower accuracy of absolute risk prediction. These models may not be appropriate for applications where absolute risk is essential for decision-making. In particular, mortality calibration differences may reflect regional variation in death rates, limiting applicability without recalibration. To enable model estimation, missing HbA1c values were imputed using random draws from published normal distributions stratified by diabetes status. While this pragmatic approach has been used in prior population-based studies, it assumes that HbA1c values in our cohort follow these external distributions and does not account for potential systematic differences related to diabetes severity, treatment intensity, or health care utilization. Consequently, the imputed HbA1c values may have introduced measurement error and could bias model performance—particularly calibration and risk estimation—for diabetes-related outcomes. Many events (e.g. medications / procedures that lead to weight loss or modify the risk of the pertinent clinical events) subsequent to the Index Date could further affect the risk of clinical outcomes evaluated in the study. However, information on these future events would not be available to the model users at the time when they are running the model, and therefore was not included in the model. Other predictive models designed for different use scenarios could incorporate information on future events to increase prediction accuracy. Patient data in the external validation dataset was fully de-identified and it is not possible to determine whether some of the patients may also have been included in the original derivation dataset. However, this overlap would likely be insubstantial, as the external validation dataset was nationwide and the derivation dataset was limited to a single state (Massachusetts) with a relatively small number of patients who have health insurance provided by United Healthcare (the primary source of data for the external validation dataset). Some of the racial / ethnic groups were not significantly represented in the dataset, and study findings may not be applicable to them. Underrepresentation of these groups could limit generalizability of the study findings. Therefore, a subgroup analysis by racial / ethnic groups could be a helpful future direction of this work. Other subgroup analyses (e.g. by sex, age and BMI categories) would also help assess generalizability of the models in more detail and should be considered for future research. Based on these analyses, models may need to be recalibrated for use in specific subgroups to ensure clinical usability. All predictive models in this study were derived and validated based on U.S. data. They may therefore not be applicable to individuals outside of the U.S., particularly in locations with significant differences in demographics, healthcare access, and obesity management strategies. BMI is not a perfect measure of excess adiposity. However, other measures of excess adiposity, such as waist circumference and body fat percentage, were not available in the data analyzed for this study and are generally not available in EHR data. As the goal of the study was to validate predictive models that could be widely used in healthcare organizations by drawing on their EHR data, focusing on BMI improves portability of the predictive models to other institutions. Weight loss due to metabolic surgery or pharmacotherapy was not considered in the analysis. The findings of the present analysis demonstrate external validity and stable predictive performance, rather than established clinical effectiveness or readiness for routine implementation. Prospective evaluation and assessment of real-world implementation outcomes will be necessary before these models can be incorporated into routine clinical practice.

## Conclusions

This study demonstrated that a set of predictive models of adverse clinical outcomes of overweight or obesity that drew upon data easily available in EHRs was generalizable to a large national cohort of U.S. patients. These findings suggest potential clinical and population health applications for utilization of predictive models of obesity-related complications in care of individual patients, population management and quality improvement initiatives. Prospective testing and real-world implementation studies would be needed before routine adoption.

## Supplementary Information

Below is the link to the electronic supplementary material.

Supplementary Material 1

## Data Availability

Datasets generated during and/or analyzed during the current study are not publicly available secondary to the institutional policies.
