# Supplementary material for: Predictive models of adverse outcomes of overweight or obesity: a nationwide cohort validation
Source: BMC Endocr Disord. 2026 Jul 10;26:265. doi: 10.1186/s12902-026-02399-4 (PMC13556077; doi:10.1186/s12902-026-02399-4)
Supplement: Supplementary file 1 [file 12902_2026_2399_MOESM1_ESM.docx]

**Supplemental Table 1.**

**ICD codes used to identify information on study predictors and outcomes.**

Supplemental Table 1A: ICD-9 / 10 codes for ASCVD

| Code | Description |
| --- | --- |
| ICD-9 Codes | |
| 410.xx | Coronary artery disease / MI |
| 411.xx | Coronary artery disease |
| 412.xx | Coronary artery disease |
| 413.xx | Coronary artery disease |
| 414.xx | Coronary artery disease |
| 429.2 | Cardiovascular Disease, Unspecified (ASCVD) |
| 433.x1 | Stroke |
| 434.x1 | Stroke |
| 437.00 | Cerebral Atherosclerosis |
| 440.xx | Peripheral vascular disease |
| 443.9 | Peripheral vascular disease |
| ICD-10 Codes | |
| I20.xx | Coronary artery disease |
| I21.xx | Coronary artery disease / MI |
| I22.xx | Coronary artery disease |
| I23.xx | Coronary artery disease |
| I24.xx | Coronary artery disease |
| I25.xx | Coronary artery disease |
| I60.xx | Stroke |
| I61.xx | Stroke |
| I62.xx | Stroke |
| I63.xx | Stroke |
| I67.2 | Cerebral Atherosclerosis |
| I70.xx | Peripheral vascular disease |
| I73.9 | Peripheral vascular disease |

Supplemental Table 1B: ICD-9 / 10 codes for heart failure

| Code | Description |
| --- | --- |
| ICD-9 Codes | |
| 402.01 | Malignant Hypertension Heart Disease with Heart Failure |
| 402.11 | Benign Hypertension Heart Disease with Heart Failure |
| 402.91 | Unspecified Hypertension Heart Disease with Heart Failure |
| 404.01 | Mal hyp ht/kd I-IV w hf |
| 404.03 | Mal hyp ht/kd stg V w hf |
| 404.11 | Hypertensive heart and renal disease, benign, with heart failure |
| 404.13 | Hypertensive heart and renal disease, benign, with heart failure and renal failure |
| 404.91 | Hypertensive heart and renal disease, unspecified, with heart failure |
| 404.93 | Hypertensive heart and renal disease, unspecified, with heart failure and renal failure |
| 428.xx | Heart failure |
| ICD-10 Codes | |
| I09.81 | Rheumatic heart failure |
| I11.0 | Hypertensive heart disease with heart failure |
| I13.0 | Hypertensive heart and chronic kidney disease with heart failure and stage 1 through stage 4 chronic kidney disease, or unspecified chronic kidney disease |
| I13.2 | Hypertensive heart and chronic kidney disease with heart failure and with stage 5 chronic kidney disease, or end stage renal disease |
| I50 | Heart failure |
| I50.xx | Heart failure |
| I97.13 | Postprocedural heart failure |
| I97.130 | Postprocedural heart failure following cardiac surgery |
| I97.131 | Postprocedural heart failure following other surgery |

Supplemental Table 1C: ICD-9 / 10 codes for type 2 diabetes mellitus

| Code | Description |
| --- | --- |
| ICD-9 Codes | |
| 250.xx | Diabetes mellitus |
| ICD-10 Codes | |
| E11.x | Diabetes mellitus type 2 |

Supplemental Table 1D: ICD-9 / 10 codes for NASH/NAFLD

| Code | Description |  |
| --- | --- | --- |
| ICD-9 Codes | | |
| 571.8 | Other chronic nonalcoholic liver disease | |
| 571.9 | Unspecified chronic liver disease without mention of alcohol | |
| ICD-10 Codes | | |
| K75.81 | other specified inflammatory liver diseases including non-alcoholic steatohepatitis | |
| K76.0 | fatty change of liver not elsewhere classified | |

Supplemental Table 1E: ICD-9 / 10 codes for sleep apnea

| Code | Description |
| --- | --- |
| ICD-9 Codes | |
| 327.2 | Organic sleep apnea |
| 780.51 | Insomnia with sleep apnea, unspecified |
| 780.53 | Hypersomnia with sleep apnea, unspecified |
| ICD-10 Codes | |
| G47.33 | Obstructive sleep apnea (adult) (pediatric) |

Supplemental Table 1F: ICD-9 / 10 codes for cancer

| Code | Description |
| --- | --- |
| ICD-9 Codes | |
| 14x.xx | Malignant Neoplasm of Lip, Oral Cavity, and Pharynx |
| 15x.xx | Malignant Neoplasm of Digestive Organs and Peritoneum |
| 16x.xx | Malignant Neoplasm of Respiratory and Intrathoracic Organs |
| 170.xx | Malignant neoplasm of bone and articular cartilage |
| 171.xx | Malignant neoplasm of connective and other soft tissue |
| 172.xx | Malignant melanoma of skin |
| 174.xx | Malignant neoplasm of female breast |
| 175.xx | Malignant neoplasm of male breast |
| 176.xx | Kaposi's sarcoma |
| 179.xx | Malignant neoplasm of uterus, part unspecified |
| 18x.xx | Malignant Neoplasm of Genitourinary Organs |
| 19x.xx | Malignant Neoplasm of Other and Unspecified Sites |
| 200.xx | Lymphosarcoma and reticulosarcoma and other specified malignant tumors of lymphatic tissue |
| 201.xx | Hodgkin's disease |
| 202.xx | Other malignant neoplasms of lymphoid and histiocytic tissue |
| 203.xx | Multiple myeloma and immunoproliferative neoplasms |
| 204.xx | Lymphoid leukemia |
| 205.xx | Myeloid leukemia |
| 206.xx | Monocytic leukemia |
| 207.xx | Other specified leukemia |
| 208.xx | Leukemia of unspecified cell type |
| 209.0x | Malignant carcinoid tumors of the small intestine |
| 209.1x | Malignant carcinoid tumors of the appendix, large intestine, and rectum |
| 209.2x | Malignant carcinoid tumors of other and unspecified sites |
| 209.3x | Malignant poorly differentiated neuroendocrine tumors |
| 230.xx | Carcinoma in situ of digestive organs |
| 231.xx | Carcinoma in situ of respiratory system |
| 233.xx | Carcinoma in situ of breast and genitourinary system |
| 234.xx | Carcinoma in situ of other and unspecified sites |
| ICD-10 Codes | |
| C00xx-D09xx | Malignant neoplasms |
| R85.614 | Cytologic evidence of malignancy on smear of anus |
| R87.614 | Cytologic evidence of malignancy on smear of cervix |
| R87.624 | Cytologic evidence of malignancy on smear of vagina |

Supplemental Table 1G: ICD-9 / 10 codes for degenerative joint disease

| Code | Description |
| --- | --- |
| ICD-9 Codes | |
| 715 | Osteoarthrosis and allied disorders |
| 715.0x | Osteoarthrosis, generalized |
| 715.1x | Osteoarthrosis, localized, primary |
| 715.2x | Osteoarthrosis, localized, secondary |
| 715.3x | Osteoarthrosis, localized, not specified whether |
| 715.8x | Osteoarthritis, multisite |
| 715.9x | Osteoarthritis, unspecified |
| ICD-10 Codes | |
| M19 | Other and unspecified osteoarthritis |
| M19.0xx | Primary osteoarthritis |
| M19.1xx | Post-traumatic osteoarthritis |
| M19.2xx | Secondary osteoarthritis |
| M19.9 | Osteoarthritis, unspecified site |
| M19.90 | Unspecified osteoarthritis, unspecified site |
| M19.91 | Primary osteoarthritis, unspecified site |
| M19.92 | Post-traumatic osteoarthritis, unspecified site |
| M19.93 | Secondary osteoarthrosis, unspecified site |

Supplemental Table 1H: ICD-9 / 10 and CPT codes for knee replacement

| Code | Description |
| --- | --- |
| ICD-9 Codes | |
| 81.54 | Total knee replacement |
| ICD-9 Procedure Codes | |
| V43.65 | Knee joint replaced by other means |
| ICD-10 Procedure Codes | |
| 0SRC07Z | Replacement of Right Knee Joint with Autologous Tissue Substitute, Open Approach |
| 0SRC0J9 | Replacement of Right Knee Joint with Synthetic Substitute, Cemented, Open Approach |
| 0SRC0JA | Replacement of Right Knee Joint with Synthetic Substitute, Uncemented, Open Approach |
| 0SRC0JZ | Replacement of Right Knee Joint with Synthetic Substitute, Open Approach |
| 0SRC0KZ | Replacement of Right Knee Joint with Nonautologous Tissue Substitute, Open Approach |
| 0SRC0L9 | Replacement of Right Knee Joint with Unicondylar Synthetic Substitute, Cemented, Open Approach |
| 0SRC0LA | Replacement of Right Knee Joint with Unicondylar Synthetic Substitute, Uncemented, Open Approach |
| 0SRC0LZ | Replacement of Right Knee Joint with Unicondylar Synthetic Substitute, Open Approach |
| 0SRD07Z | Replacement of Left Knee Joint with Autologous Tissue Substitute, Open Approach |
| 0SRD0J9 | Replacement of Left Knee Joint with Synthetic Substitute, Cemented, Open Approach |
| 0SRD0JA | Replacement of Left Knee Joint with Synthetic Substitute, Uncemented, Open Approach |
| 0SRD0JZ | Replacement of Left Knee Joint with Synthetic Substitute, Open Approach |
| 0SRD0KZ | Replacement of Left Knee Joint with Nonautologous Tissue Substitute, Open Approach |
| 0SRD0L9 | Replacement of Left Knee Joint with Unicondylar Synthetic Substitute, Cemented, Open Approach |
| 0SRD0LA | Replacement of Left Knee Joint with Unicondylar Synthetic Substitute, Uncemented, Open Approach |
| 0SRD0LZ | Replacement of Left Knee Joint with Unicondylar Synthetic Substitute, Open Approach |
| 0SRT07Z | Replacement of Right Knee Joint, Femoral Surface with Autologous Tissue Substitute, Open Approach |
| 0SRT0J9 | Replacement of Right Knee Joint, Femoral Surface with Synthetic Substitute, Cemented, Open Approach |
| 0SRT0JA | Replacement of Right Knee Joint, Femoral Surface with Synthetic Substitute, Uncemented, Open Approach |
| 0SRT0JZ | Replacement of Right Knee Joint, Femoral Surface with Synthetic Substitute, Open Approach |
| 0SRT0KZ | Replacement of Right Knee Joint, Femoral Surface with Nonautologous Tissue Substitute, Open Approach |
| 0SRU07Z | Replacement of Left Knee Joint, Femoral Surface with Autologous Tissue Substitute, Open Approach |
| 0SRU0J9 | Replacement of Left Knee Joint, Femoral Surface with Synthetic Substitute, Cemented, Open Approach |
| 0SRU0JA | Replacement of Left Knee Joint, Femoral Surface with Synthetic Substitute, Uncemented, Open Approach |
| 0SRU0JZ | Replacement of Left Knee Joint, Femoral Surface with Synthetic Substitute, Open Approach |
| 0SRU0KZ | Replacement of Left Knee Joint, Femoral Surface with Nonautologous Tissue Substitute, Open Approach |
| 0SRV07Z | Replacement of Right Knee Joint, Tibial Surface with Autologous Tissue Substitute, Open Approach |
| 0SRV0J9 | Replacement of Right Knee Joint, Tibial Surface with Synthetic Substitute, Cemented, Open Approach |
| 0SRV0JA | Replacement of Right Knee Joint, Tibial Surface with Synthetic Substitute, Uncemented, Open Approach |
| 0SRV0JZ | Replacement of Right Knee Joint, Tibial Surface with Synthetic Substitute, Open Approach |
| 0SRV0KZ | Replacement of Right Knee Joint, Tibial Surface with Nonautologous Tissue Substitute, Open Approach |
| 0SRW07Z | Replacement of Left Knee Joint, Tibial Surface with Autologous Tissue Substitute, Open Approach |
| 0SRW0J9 | Replacement of Left Knee Joint, Tibial Surface with Synthetic Substitute, Cemented, Open Approach |
| 0SRW0JA | Replacement of Left Knee Joint, Tibial Surface with Synthetic Substitute, Uncemented, Open Approach |
| 0SRW0JZ | Replacement of Left Knee Joint, Tibial Surface with Synthetic Substitute, Open Approach |
| 0SRW0KZ | Replacement of Left Knee Joint, Tibial Surface with Nonautologous Tissue Substitute, Open Approach |
| CPT Codes | |
| 01402 | Anesthesia for open procedures on knee joint; total knee replacement |
| 0396T | Intra-operative use of kinetic balance sensor for implant stability during knee replacement arthroplasty (List separately in addition to code for primary procedure) |
| 27447 | Arthroplasty, knee, condyle and plateau; medial AND lateral compartments with or without patella resurfacing (total knee replacement) |

Supplemental Table 1I: ICD-9 / 10 codes for obesity-related cancer

| Code | Description | Only Used for History | Female Only Cancer |
| --- | --- | --- | --- |
| ICD-9 Codes | | | |
| 150 | Malignant neoplasm of esophagus |  |  |
| 150.0 | Malignant neoplasm of cervical esophagus |  |  |
| 150.1 | Malignant neoplasm of thoracic esophagus |  |  |
| 150.2 | Malignant neoplasm of abdominal esophagus |  |  |
| 150.3 | Malignant neoplasm of upper third of esophagus |  |  |
| 150.4 | Malignant neoplasm of middle third of esophagus |  |  |
| 150.5 | Malignant neoplasm of lower third of esophagus |  |  |
| 150.8 | Malignant neoplasm of other specified part of esophagus |  |  |
| 150.9 | Malignant neoplasm of esophagus, unspecified |  |  |
| 151.0 | Malignant neoplasm of cardia |  |  |
| 151.1 | Malignant neoplasm of pylorus |  |  |
| 151.2 | Malignant neoplasm of pyloric antrum |  |  |
| 151.3 | Malignant neoplasm of fundus of stomach |  |  |
| 151.4 | Malignant neoplasm of body of stomach |  |  |
| 151.5 | Malignant neoplasm of lesser curvature of stomach, unspecified |  |  |
| 151.6 | Malignant neoplasm of greater curvature of stomach, unspecified |  |  |
| 151.8 | Malignant neoplasm of other specified sites of stomach |  |  |
| 151.9 | Malignant neoplasm of stomach, unspecified |  |  |
| 153 | Malignant neoplasm of colon |  |  |
| 153.0 | Malignant neoplasm of hepatic flexure |  |  |
| 153.1 | Malignant neoplasm of transverse colon |  |  |
| 153.2 | Malignant neoplasm of descending colon |  |  |
| 153.3 | Malignant neoplasm of sigmoid colon |  |  |
| 153.4 | Malignant neoplasm of cecum |  |  |
| 153.5 | Malignant neoplasm of appendix vermiformis |  |  |
| 153.6 | Malignant neoplasm of ascending colon |  |  |
| 153.7 | Malignant neoplasm of splenic flexure |  |  |
| 153.8 | Malignant neoplasm of other specified sites of large intestine |  |  |
| 153.9 | Malignant neoplasm of colon, unspecified |  |  |
| 154 | Malignant neoplasm of rectum, rectosigmoid junction, and anus |  |  |
| 154.0 | Malignant neoplasm of rectosigmoid junction |  |  |
| 154.1 | Malignant neoplasm of rectum |  |  |
| 154.2 | Malignant neoplasm of anal canal |  |  |
| 154.3 | Malignant neoplasm of anus, unspecified |  |  |
| 154.8 | Malignant neoplasm of other sites of rectum, rectosigmoid junction, and anus |  |  |
| 155 | Malignant neoplasm of liver and intrahepatic bile ducts |  |  |
| 155.0 | Malignant neoplasm of liver, primary |  |  |
| 155.1 | Malignant neoplasm of intrahepatic bile ducts |  |  |
| 155.2 | Malignant neoplasm of liver, not specified as primary or secondary |  |  |
| 156.8 | Malignant neoplasm of other specified sites of gallbladder and extrahepatic bile ducts |  |  |
| 156.9 | Malignant neoplasm of biliary tract, part unspecified |  |  |
| 157 | Malignant neoplasm of pancreas |  |  |
| 157.0 | Malignant neoplasm of head of pancreas |  |  |
| 157.1 | Malignant neoplasm of body of pancreas |  |  |
| 157.2 | Malignant neoplasm of tail of pancreas |  |  |
| 157.3 | Malignant neoplasm of pancreatic duct |  |  |
| 157.4 | Malignant neoplasm of islets of Langerhans |  |  |
| 157.8 | Malignant neoplasm of other specified sites of pancreas |  |  |
| 157.9 | Malignant neoplasm of pancreas, part unspecified |  |  |
| 174 | Malignant neoplasm of female breast |  | √ |
| 174.0 | Malignant neoplasm of nipple and areola of female breast |  | √ |
| 174.1 | Malignant neoplasm of central portion of female breast |  | √ |
| 174.2 | Malignant neoplasm of upper-inner quadrant of female breast |  | √ |
| 174.3 | Malignant neoplasm of lower-inner quadrant of female breast |  | √ |
| 174.4 | Malignant neoplasm of upper-outer quadrant of female breast |  | √ |
| 174.5 | Malignant neoplasm of lower-outer quadrant of female breast |  | √ |
| 174.6 | Malignant neoplasm of axillary tail of female breast |  | √ |
| 174.8 | Malignant neoplasm of other specified sites of female breast |  | √ |
| 174.9 | Malignant neoplasm of breast (female), unspecified |  | √ |
| 179 | Malignant neoplasm of uterus, part unspecified |  | √ |
| 179.0 | Malignant neoplasm of uterus, part unspecified |  | √ |
| 182 | Malignant neoplasm of body of uterus |  | √ |
| 182.0 | Malignant neoplasm of corpus uteri, except isthmus |  | √ |
| 182.1 | Malignant neoplasm of isthmus |  | √ |
| 182.8 | Malignant neoplasm of other specified sites of body of uterus |  | √ |
| 183.0 | Malignant neoplasm of ovary |  | √ |
| 189.0 | Malignant neoplasm of kidney, except pelvis |  |  |
| 192.1 | Malignant neoplasm of cerebral meninges |  |  |
| 192.3 | Malignant neoplasm of spinal meninges |  |  |
| 203 | Multiple myeloma and immunoproliferative neoplasms |  |  |
| 203.0 | Multiple myeloma |  |  |
| 203.00 | Multiple myeloma without mention of remission |  |  |
| 203.01 | Multiple myeloma in remission |  |  |
| 203.02 | Multiple myeloma, in relapse |  |  |
| 203.1 | Plasma cell leukemia |  |  |
| 203.10 | Plasma cell leukemia without mention of remission |  |  |
| 203.11 | Plasma cell leukemia in remission |  |  |
| 203.12 | Plasma cell leukemia, in relapse |  |  |
| 203.8 | Other immunoproliferative neoplasms |  |  |
| 203.80 | Other immunoproliferative neoplasms without mention of remission |  |  |
| 203.81 | Other immunoproliferative neoplasms in remission |  |  |
| 225.2 | Benign neoplasm of cerebral meninges |  |  |
| 225.4 | Benign neoplasm of spinal meninges |  |  |
| V10.03 | Personal history of malignant neoplasm of esophagus | √ |  |
| V10.04 | Personal history of malignant neoplasm of stomach | √ |  |
| V10.05 | Personal history of malignant neoplasm of large intestine | √ |  |
| V10.06 | Personal history of malignant neoplasm of rectum, rectosigmoid junction, and anus | √ |  |
| V10.07 | Personal history of malignant neoplasm of liver | √ |  |
| V10.09 | Personal history of malignant neoplasm of other sites in gastrointestinal tract | √ |  |
| V10.3 | Personal history of malignant neoplasm of breast | √ | √ |
| V10.43 | Personal history of malignant neoplasm of ovary | √ | √ |
| V10.52 | Personal history of malignant neoplasm of kidney | √ |  |
| ICD-10 Codes | | | |
| C15.3 | Malignant neoplasm of upper third of esophagus |  |  |
| C15.4 | Malignant neoplasm of middle third of esophagus |  |  |
| C15.5 | Malignant neoplasm of lower third of esophagus |  |  |
| C15.8 | Malignant neoplasm of overlapping sites of esophagus |  |  |
| C15.9 | Malignant neoplasm of esophagus, unspecified |  |  |
| C16.0 | Malignant neoplasm of cardia |  |  |
| C16.1 | Malignant neoplasm of fundus of stomach |  |  |
| C16.2 | Malignant neoplasm of body of stomach |  |  |
| C16.3 | Malignant neoplasm of pyloric antrum |  |  |
| C16.4 | Malignant neoplasm of pylorus |  |  |
| C16.5 | Malignant neoplasm of lesser curvature of stomach, unspecified |  |  |
| C16.6 | Malignant neoplasm of greater curvature of stomach, unspecified |  |  |
| C16.8 | Malignant neoplasm of overlapping sites of stomach |  |  |
| C16.9 | Malignant neoplasm of stomach, unspecified |  |  |
| C18.0 | Malignant neoplasm of cecum |  |  |
| C18.1 | Malignant neoplasm of appendix |  |  |
| C18.2 | Malignant neoplasm of ascending colon |  |  |
| C18.3 | Malignant neoplasm of hepatic flexure |  |  |
| C18.4 | Malignant neoplasm of transverse colon |  |  |
| C18.5 | Malignant neoplasm of splenic flexure |  |  |
| C18.6 | Malignant neoplasm of descending colon |  |  |
| C18.7 | Malignant neoplasm of sigmoid colon |  |  |
| C18.8 | Malignant neoplasm of overlapping sites of colon |  |  |
| C18.9 | Malignant neoplasm of colon, unspecified |  |  |
| C19 | Malignant neoplasm of rectosigmoid junction |  |  |
| C20 | Malignant neoplasm of rectum |  |  |
| C21.8 | Malignant neoplasm of overlapping sites of rectum, anus and anal canal |  |  |
| C22.0 | Liver cell carcinoma |  |  |
| C22.1 | Intrahepatic bile duct carcinoma |  |  |
| C22.7 | Other specified carcinomas of liver |  |  |
| C22.8 | Malignant neoplasm of liver, primary, unspecified as to type |  |  |
| C22.9 | Malignant neoplasm of liver, not specified as primary or secondary |  |  |
| C23 | Malignant neoplasm of gallbladder |  |  |
| C24.0 | Malignant neoplasm of extrahepatic bile duct |  |  |
| C24.1 | Malignant neoplasm of ampulla of vater |  |  |
| C24.8 | Malignant neoplasm of overlapping sites of biliary tract |  |  |
| C24.9 | Malignant neoplasm of biliary tract, unspecified |  |  |
| C25.0 | Malignant neoplasm of head of pancreas |  |  |
| C25.1 | Malignant neoplasm of body of pancreas |  |  |
| C25.2 | Malignant neoplasm of tail of pancreas |  |  |
| C25.3 | Malignant neoplasm of pancreatic duct |  |  |
| C25.4 | Malignant neoplasm of endocrine pancreas |  |  |
| C25.7 | Malignant neoplasm of other parts of pancreas |  |  |
| C25.8 | Malignant neoplasm of overlapping sites of pancreas |  |  |
| C25.9 | Malignant neoplasm of pancreas, unspecified |  |  |
| C50.011 | Malignant neoplasm of nipple and areola, right female breast |  | √ |
| C50.012 | Malignant neoplasm of nipple and areola, left female breast |  | √ |
| C50.019 | Malignant neoplasm of nipple and areola, unspecified female breast |  | √ |
| C50.111 | Malignant neoplasm of central portion of right female breast |  | √ |
| C50.112 | Malignant neoplasm of central portion of left female breast |  | √ |
| C50.119 | Malignant neoplasm of central portion of unspecified female breast |  | √ |
| C50.211 | Malignant neoplasm of upper-inner quadrant of right female breast |  | √ |
| C50.212 | Malignant neoplasm of upper-inner quadrant of left female breast |  | √ |
| C50.219 | Malignant neoplasm of upper-inner quadrant of unspecified female breast |  | √ |
| C50.311 | Malignant neoplasm of lower-inner quadrant of right female breast |  | √ |
| C50.312 | Malignant neoplasm of lower-inner quadrant of left female breast |  | √ |
| C50.319 | Malignant neoplasm of lower-inner quadrant of unspecified female breast |  | √ |
| C50.411 | Malignant neoplasm of upper-outer quadrant of right female breast |  | √ |
| C50.412 | Malignant neoplasm of upper-outer quadrant of left female breast |  | √ |
| C50.419 | Malignant neoplasm of upper-outer quadrant of unspecified female breast |  | √ |
| C50.511 | Malignant neoplasm of lower-outer quadrant of right female breast |  | √ |
| C50.512 | Malignant neoplasm of lower-outer quadrant of left female breast |  | √ |
| C50.519 | Malignant neoplasm of lower-outer quadrant of unspecified female breast |  | √ |
| C50.611 | Malignant neoplasm of axillary tail of right female breast |  | √ |
| C50.612 | Malignant neoplasm of axillary tail of left female breast |  | √ |
| C50.619 | Malignant neoplasm of axillary tail of unspecified female breast |  | √ |
| C50.811 | Malignant neoplasm of overlapping sites of right female breast |  | √ |
| C50.812 | Malignant neoplasm of overlapping sites of left female breast |  | √ |
| C50.819 | Malignant neoplasm of overlapping sites of unspecified female breast |  | √ |
| C50.911 | Malignant neoplasm of unspecified site of right female breast |  | √ |
| C50.912 | Malignant neoplasm of unspecified site of left female breast |  | √ |
| C50.919 | Malignant neoplasm of unspecified site of unspecified female breast |  | √ |
| C54.0 | Malignant neoplasm of isthmus uteri |  | √ |
| C54.1 | Malignant neoplasm of endometrium |  | √ |
| C54.2 | Malignant neoplasm of myometrium |  | √ |
| C54.3 | Malignant neoplasm of fundus uteri |  | √ |
| C54.8 | Malignant neoplasm of overlapping sites of corpus uteri |  | √ |
| C54.9 | Malignant neoplasm of corpus uteri, unspecified |  | √ |
| C55 | Malignant neoplasm of uterus, part unspecified |  | √ |
| C56.1 | Malignant neoplasm of right ovary |  | √ |
| C56.2 | Malignant neoplasm of left ovary |  | √ |
| C56.9 | Malignant neoplasm of unspecified ovary |  | √ |
| C64.1 | Malignant neoplasm of right kidney, except renal pelvis |  |  |
| C64.2 | Malignant neoplasm of left kidney, except renal pelvis |  |  |
| C64.9 | Malignant neoplasm of unspecified kidney, except renal pelvis |  |  |
| C70.0 | Malignant neoplasm of cerebral meninges |  |  |
| C70.1 | Malignant neoplasm of spinal meninges |  |  |
| C70.9 | Malignant neoplasm of meninges, unspecified |  |  |
| C90.00 | Multiple myeloma not having achieved remission |  |  |
| C90.01 | Multiple myeloma in remission |  |  |
| C90.02 | Multiple myeloma in relapse |  |  |
| C90.10 | Plasma cell leukemia not having achieved remission |  |  |
| C90.11 | Plasma cell leukemia in remission |  |  |
| C90.12 | Plasma cell leukemia in relapse |  |  |
| C90.20 | Extramedullary plasmacytoma not having achieved remission |  |  |
| C90.21 | Extramedullary plasmacytoma in remission |  |  |
| C90.22 | Extramedullary plasmacytoma in relapse |  |  |
| C90.30 | Solitary plasmacytoma not having achieved remission |  |  |
| C90.31 | Solitary plasmacytoma in remission |  |  |
| C90.32 | Solitary plasmacytoma in relapse |  |  |
| D32.0 | Benign neoplasm of cerebral meninges |  |  |
| D32.1 | Benign neoplasm of spinal meninges |  |  |
| D32.9 | Benign neoplasm of meninges, unspecified |  |  |
| D42.0 | Neoplasm of uncertain behavior of cerebral meninges |  |  |
| D42.1 | Neoplasm of uncertain behavior of spinal meninges |  |  |
| D42.9 | Neoplasm of uncertain behavior of meninges, unspecified |  |  |

Supplemental Table 1J: ICD-9 / 10 codes for chronic kidney disease

| Code | Description |
| --- | --- |
| ICD-9 Codes | |
| 585.6 | End stage renal disease |
| 403.x1 | Hypertensive chronic kidney disease, malignant, with chronic kidney disease stage V or end stage renal disease |
| 404.x2 | Hypertensive heart and chronic kidney disease, malignant, without heart failure and with chronic kidney disease stage V or end stage renal disease |
| 404.x3 | Hypertensive heart and chronic kidney disease, malignant, with heart failure and with chronic kidney disease stage V or end stage renal disease |
| 996.81 | Complications of transplanted kidney |
| ICD-9 Procedure Codes | |
| V42.0 | Kidney replaced by transplant |
| 5569 | Other kidney transplantation |
| 5553 | Rejected kidney nephrectomy |
| ICD-10 Codes | |
| Z49.xx | Encounter for care involving renal dialysis |
| N18.6 | End stage renal disease |
| I12.0 | Hypertensive chronic kidney disease with stage 5 chronic kidney disease or end stage renal disease |
| I13.11 | Hypertensive heart and chronic kidney disease with stage 5 chronic kidney disease, or end stage renal disease |
| Z94.0 | Kidney transplant status |
| T86.1x | Complications of kidney transplant |
| ICD-10 Procedure Codes | |
| 0TY10Z0 | Transplantation of Left Kidney, Allogeneic, Open Approach |
| 0TY00Z0 | Transplantation of Right Kidney, Allogeneic, Open Approach |
| 0TY10Z1 | Transplantation of Left Kidney, Syngeneic, Open Approach |
| 0TY00Z1 | Transplantation of Right Kidney, Syngeneic, Open Approach |
| CPT Procedure Codes | |
| 50360 | Renal allotransplantation, implantation of graft; excluding donor and recipient nephrectomy |
| 50365 | Renal allotransplantation, implantation of graft; with recipient nephrectomy |
| 76776 | Ultrasound, transplanted kidney, real time and duplex Doppler with image documentation |

Supplemental Table 1K: ICD-9 / 10 codes for Risk Factors included in analysis

| Code | Code Type | Description |
| --- | --- | --- |
| Hypertension | | |
| 401.xx | ICD-9 | Hypertension |
| 402.xx | ICD-9 | Hypertensive heart disease |
| 403.xx | ICD-9 | Hypertensive renal disease |
| 404.xx | ICD-9 | Hypertensive heart and renal disease |
| 405.xx | ICD-9 | Secondary hypertension |
| 437.2 | ICD-9 | Hypertensive encephalopathy |
| I10 | ICD-10 | Hypertension |
| I11.x | ICD-10 | Hypertensive heart disease |
| I12.x | ICD-10 | Hypertensive chronic kidney disease |
| I13.xx | ICD-10 | Hypertensive heart disease and chronic kidney disease |
| I15.x | ICD-10 | Secondary hypertension |
| I16.x | ICD-10 | Hypertensive crisis |
| Hepatitis B | | |
| 070.2 | ICD-9 | Viral hepatitis B with hepatic coma |
| 070.2x | ICD-9 | Viral hepatitis B with hepatic coma |
| 070.3 | ICD-9 | Viral hepatitis B without mention of hepatic coma |
| 070.3x | ICD-9 | Viral hepatitis B without mention of hepatic coma |
| V02.61 | ICD-9 | Hepatitis B carrier |
| B16 | ICD-10 | Acute hepatitis B |
| B16.x | ICD-10 | Acute hepatitis B |
| B18.0 | ICD-10 | Chronic viral hepatitis B |
| B18.1 | ICD-10 | Chronic viral hepatitis B |
| B19.1x | ICD-10 | Unspecified viral hepatitis B |
| Z22.51 | ICD-10 | Carrier of viral hepatitis B |
| Hepatitis C | | |
| 070.44 | ICD-9 | Chronic hepatitis C with hepatic coma |
| 070.54 | ICD-9 | Chronic hepatitis C without mention of hepatic coma |
| 070.7 | ICD-9 | Unspecified viral hepatitis C |
| 070.70 | ICD-9 | Unspecified viral hepatitis C |
| 070.71 | ICD-9 | Unspecified viral hepatitis C |
| V02.62 | ICD-9 | Hepatitis C carrier |
| B17.10 | ICD-10 | Acute hepatitis C |
| B17.11 | ICD-10 | Acute hepatitis C |
| B18.2 | ICD-10 | Chronic viral hepatitis C |
| B19.20 | ICD-10 | Unspecified viral hepatitis C |
| B19.21 | ICD-10 | Unspecified viral hepatitis C |
| Z22.52 | ICD-10 | Carrier of viral hepatitis C |
| Chronic Inflammation Diseases | | |
| 041.86 | ICD-9 | Helicobacter pylori [H. pylori] infection in conditions classified elsewhere and of unspecified site |
| 556.x | ICD-9 | Ulcerative colitis |
| 714.xx | ICD-9 | Rheumatoid arthritis and other inflammatory polyarthropathies |
| 340 | ICD-9 | Multiple sclerosis |
| 710.0 | ICD-9 | Systemic lupus erythematosus (SLE) |
| B96.81 | ICD10 | Helicobacter pylori [H. pylori] as the cause of diseases classified elsewhere |
| G35 | ICD-10 | Multiple sclerosis |
| K50.xxx | ICD-10 | Chron’s disease |
| K51.xx | ICD-10 | Ulcerative colitis, unspecified, without complications |
| M05.xxx | ICD-10 | Rheumatoid arthritis |
| M06.xxx | ICD-10 | Other rheumatoid arthritis |
| M08.0xx | ICD-10 | Juvenile rheumatoid arthritis |
| M08.2xx | ICD-10 | Juvenile rheumatoid arthritis |
| M08.3 | ICD-10 | Juvenile rheumatoid polyarthritis (seronegative) |
| M08.4xx | ICD-10 | Pauciarticular juvenile rheumatoid arthritis |
|  |  |  |
| M32.xx | ICD-10 | Systemic lupus erythematosus (SLE) |
|  | Knee injury and/or surgery, including meniscectomy, ICD9/10, and CPT codes | |
| 836 | ICD-9 | Dislocation of knee |
| 836.0 | ICD-9 | Tear of medial cartilage or meniscus of knee, current |
| 836.1 | ICD-9 | Tear of lateral cartilage or meniscus of knee, current |
| 836.2 | ICD-9 | Tear meniscus NEC-curren |
| 836.3 | ICD-9 | Dislocation of patella, closed |
| 836.4 | ICD-9 | Dislocation patella-open |
| 836.5x | ICD-9 | Other dislocation of knee, closed |
| 836.6x | ICD-9 | Other dislocation of knee, open |
| 844.x | ICD-9 | Sprain of knee |
| 959.7 | ICD-9 | Other and unspecified injury to knee, leg, ankle, and foot |
| S80 | ICD-10 | Superficial injury of knee and lower leg |
| S80.0xxx | ICD-10 | Contusion of knee |
| S82.0xxx | ICD-10 | Fracture of patella |
| S83.0xxx | ICD-10 | Dislocation and sprain of joints and ligaments of knee |
| S83.1xxx | ICD-10 | Subluxation and dislocation of knee |
| S83.2xx | ICD-10 | Tear of meniscus |
| S83.3xxx | ICD-10 | Tear of articular cartilage of knee, current |
| S83.4xxx | ICD-10 | Sprain of collateral ligament of knee |
| S83.5xxx | ICD-10 | Sprain of cruciate ligament of knee |
| S83.6xxx | ICD-10 | Sprain of the superior tibiofibular joint and ligament |
| S83.8xxx | ICD-10 | Sprain of other specified parts of knee |
| S83.9xxx | ICD-10 | Sprain of unspecified site of knee |
| 27332 | CPT | Arthrotomy, with excision of semilunar cartilage (meniscectomy) knee; medial OR lateral |
| 27333 | CPT | Arthrotomy, with excision of semilunar cartilage (meniscectomy) knee; medial AND lateral |
| 27403 | CPT | Arthrotomy with meniscus repair, knee |
| 27405 | CPT | Repair, primary, torn ligament and/or capsule, knee; collateral |
| 27407 | CPT | Repair, primary, torn ligament and/or capsule, knee; cruciate |
| 27409 | CPT | Repair, primary, torn ligament and/or capsule, knee; collateral and cruciate ligaments |
| 27412 | CPT | Inplantations and autografts of the knee |
| 27415 | CPT | Inplantations and autografts of the knee |
| 27418 | CPT | Inplantations and autografts of the knee |
| 27420 | CPT | Knee and Kneecap revision |
| 27422 | CPT | Knee and Kneecap revision |
| 27424 | CPT | Knee and Kneecap revision |
| 27425 | CPT | Knee and Kneecap revision |
| 27427 | CPT | Knee and Kneecap revision |
| 27428 | CPT | Knee and Kneecap revision |
| 27429 | CPT | Knee and Kneecap revision |
| 27440-27447 | CPT | Revision of knee joint, total knee replacement |
| 27570 | CPT | Manipulation of knee joint under general anesthesia (includes application of traction or other fixation devices) |
| 29866 | CPT | Arthroscopy, knee, surgical; osteochondral autograft(s) (eg, mosaicplasty) (includes harvesting of the autograft[s]) |
| 29867 | CPT | Arthroscopy, knee, surgical; osteochondral allograft (eg, mosaicplasty) |
| 29868 | CPT | Arthroscopy, knee, surgical; meniscal transplantation (includes arthrotomy for meniscal insertion), medial or lateral |
| 29870, 29871, 29873-29877, 29879 | CPT | Knee arthroscopy |
| 29880 | CPT | Arthroscopy, knee, surgical; with meniscectomy (medial AND lateral, including any meniscal shaving) including debridement/shaving of articular cartilage (chondroplasty), same or separate compartment(s), when performed |
| 29881 | CPT | Arthroscopy, knee, surgical; with meniscectomy (medial OR lateral, including any meniscal shaving) including debridement/shaving of articular cartilage (chondroplasty), same or separate compartment(s), when performed |
| 29882 | CPT | Arthroscopy, knee, surgical; with meniscus repair (medial OR lateral) |
| 29883 | CPT | Arthroscopy, knee, surgical; with meniscus repair (medial AND lateral) |
| 29884-29889 | CPT | Arthroscopy, knee, surgical; with lysis of adhesions, with or without manipulation (separate procedure) And cruciate ligament repair/augmentation |
| G0289 | CPT | Arthroscopy, knee, surgical, for removal of loose body, foreign body, debridement/shaving of articular cartilage (chrondroplasty) at the time of other surgical knee arthroscopy in a different compartment of the same knee |
|  | Valvular Heart Disease | |
| 424.xx | ICD-9 | Heart valve disorders and endocarditis |
| I34.x | ICD-10 | Mitral valve disorders |
| I35.x | ICD-10 | Nonrheumatic aortic valve disorders |
| I36.x | ICD-10 | Nonrheumatic tricuspid valve disorders |
|  | Alcohol Abuse | |
| 94.46 | ICD-9 | Alcoholism counselling |
| 94.53 | ICD-9 | Referral for alcoholism rehabilitation |
| 94.6 | ICD-9 | Alcohol and drug rehabilitation and detoxification |
| 94.61 | ICD-9 | Alcohol rehabilitation |
| 94.62 | ICD-9 | Alcohol detoxification |
| 94.63 | ICD-9 | Alcohol rehabilitation and detoxification |
| 94.67 | ICD-9 | Combined alcohol and drug rehabilitation |
| 94.68 | ICD-9 | Combined alcohol and drug detoxification |
| 94.69 | ICD-9 | Combined alcohol and drug rehabilitation and detoxification |
| 291.xx | ICD-9 | Alcoholic psychoses |
| 303.xx | ICD-9 | Alcohol dependence syndrome |
| 305.0x | ICD-9 | Alcohol abuse |
| 571.0 | ICD-9 | Alcoholic fatty liver |
| 571.1 | ICD-9 | Acute alcoholic hepatitis |
| 571.2 | ICD-9 | Alcoholic cirrhosis of liver |
| 571.3 | ICD-9 | Alcoholic liver damage, unspecified |
| V11.3 | ICD-9 | Personal history of alcoholism |
| F10.1xx | ICD-10 | Alcohol abuse |
| F10.2xx | ICD-10 | Alcohol dependence |
| Z71.4 | ICD-10 | Alcohol abuse counseling and surveillance |
| K70.xx | ICD-10 | Alcoholic liver disease |
| K85.2x | ICD-10 | Alcohol induced acute pancreatitis |
| K86.0 | ICD-10 | Alcohol-induced chronic pancreatitis |
| Z71.41 | ICD-10 | Alcohol abuse counseling and surveillance of alcoholic |

Supplemental Table 2: Data Sources for Outcomes and Risk Factors

| **Outcome** | **Ascertainment Sources** |
| --- | --- |
| ASCVD | ICD-9, ICD-10 diagnosis codes* |
| Type 2 Diabetes Mellitus | ICD-9, ICD-10 diagnosis codes |
| NASH/NAFLD | ICD-9, ICD-10 diagnosis codes |
| Sleep Apnea | ICD-9, ICD-10 diagnosis codes |
| Cancer | ICD-9, ICD-10 diagnosis codes |
| Degenerative Joint Disease | ICD-9, ICD-10 diagnosis codes |
| Knee Replacement | ICD-9 diagnosis codes,  ICD-9, ICD-10 procedure codes,  CPT codes |
| Obesity-Related Cancer | ICD-9, ICD-10 diagnosis codes |
| Chronic Kidney Disease | ICD-9, ICD-10 diagnosis codes,  ICD-9, ICD-10 procedure codes,  CPT codes |
| Hypertension | ICD-9, ICD-10 diagnosis codes  Blood pressure measurements ≥ 140/90 mm Hg |
| Hepatitis B | ICD-9, ICD-10 diagnosis codes |
| Hepatitis C | ICD-9, ICD-10 diagnosis codes |
| Chronic Inflammation Diseases | ICD-9, ICD-10 diagnosis codes |
| Knee Injury and/or Surgery | ICD-9, ICD-10 diagnosis codes,  CPT codes |
| Valvular Heart Disease | ICD-9, ICD-10 diagnosis codes |
| Alcohol Abuse | ICD-9, ICD-10 diagnosis codes |
| Smoking Status | Electronic medical record entries |
| eGFR | Laboratory measurements |
| HbA1c | Laboratory measurements |
| Family History of diabetes, cancer, and ASCVD variables | ICD-9, ICD-10 diagnosis codes  Electronic medical record entries |
| LDL cholesterol | Laboratory measurements |
| Proteinuria | Laboratory measurements (urine albumin to creatinine ratio ≥ 30 mg/g) |
| Renal replacement | ICD-9, ICD-10 diagnosis codes |

* Diagnosis codes identified in both claims and encounter documentation
